# Scaphocapitate fusion in stage III Kienböck’s disease: effects of lunarectomy on postoperative pain and function

**DOI:** 10.1007/s00402-025-05783-2

**Published:** 2025-03-04

**Authors:** Amr Elshahhat, Khaled Nour, Yasser Abed

**Affiliations:** Mansoura university, Mansoura, Egypt

**Keywords:** Kienböck’s disease, Scaphocapitate fusion, Lunarectomy, Carpal collapse, Radio-scaphoid angle, Carpal height ratio

## Abstract

**Background:**

Kienböck’s disease (KD) is a progressive condition characterized by avascular necrosis of the lunate, leading to carpal collapse and wrist dysfunction. Scaphocapitate fusion (SCF) is a salvage procedure often used in advanced stages of KD to stabilize the wrist and alleviate symptoms. This study aimed to evaluate the clinical and radiological outcomes of SCF in stage III KD, with a specific focus on the impact of lunarectomy on postoperative pain and function.

**Materials and methods:**

This retrospective study included 25 patients with stage III KD who received SCF treatment and were followed between December 2019 and August 2024. Patients were categorized into groups of lunarectomy (*n* = 14) and lunate preservation (*n* = 11). Clinical outcomes were assessed using grip strength, range of motion (ROM), Visual Analog Scale (VAS) for pain, Modified Mayo Wrist Score (MMWS), and Disabilities of the Arm, Shoulder, and Hand (DASH) score. Radiological evaluations included measurements of carpal alignment and fusion success.

**Results:**

SCF significantly improved grip strength, ROM, and pain levels across all patients (*p* < 0.05). Patients with lunarectomy showed greater improvements in combined flexion-extension ROM (*p* = 0.001), grip strength (*p* = 0.0023), MMWS (*p* = 0.0033), and pain reduction (*p* = 0.037) compared to those with lunate preservation. Fusion was achieved in 92% of patients, with no significant differences in radiological outcomes between the two groups.

**Conclusion:**

SCF is an effective intervention for Stage III KD, offering pain relief and functional improvement. Lunarectomy may provide advantages over lunate preservation, particularly in terms of pain reduction and ROM, making it a considerable option for the surgical management of advanced KD.

**Level of evidence:**

level IV.

## Introduction

Kienböck’s disease (KD) is generally caused by a disruption in blood flow to the lunate. Repetitive stress, an unusual load on lunate, a negative ulnar variance, or a decreased radial inclination are possible contributing factors. Due to persistent pathological loads, untreated ischemia leads to weakening, compression, and vertical collapse of the lunate [1]. The modified Lichtman classification (Table 1) provides a four-stage framework for categorizing KD based on radiographic disease progression, ranging from normal findings to lunate sclerosis, lunate collapse, and ultimately peri-lunate arthritis [2]. A key distinction between stage IIIA and IIIB lies in scaphoid flexion and carpal malalignment. In stage IIIC, there is an additional coronally oriented lunate fracture, which can sometimes lead to misdiagnosis as an isolated lunate fracture, particularly in patients with a relevant traumatic history [3]. While radiographs remain the primary imaging modality for diagnosing KD, they lack sensitivity in early stages. ***Schmitt et al.*** introduced a magnetic resonance imaging (MRI)-based classification system that evaluates lunate perfusion, categorizing the disease into four main types: (N) normal, (A) ischemic, (B) partially necrotic (proximal, middle, or distal zones), and (C) necrotic with poor viability. This system offers valuable insights into the vascular status of the lunate, which may influence treatment decisions [2, 4].

**Table 1:** The modified Lichtman classification, and the Bain arthroscopic grading system for KD [2]

**The modified Lichtman classification (Osseous classification)**	
Stage I	No radiographic evidence of bony changes, changes seen on MRI only
Stage II	Sclerosis of lunate
Stage III IIIA IIIB IIIC	Lunate collapse and fragmentation Maintained carpal alignment (No scaphoid rotation) With associated carpal collapse (Fixed scaphoid rotation) With associated coronally oriented lunate fracture
Stage IV	Evidence of advanced carpal collapse with peri-lunate carpal arthrosis
**Bain arthroscopic grading (Intermediate column articular surface)**	
Grade 0	No nonfunctional articular surfaces
Grade 1	One nonfunctional surface (Proximal lunate)
Grade 2a Grade 2b	Two nonfunctional surfaces (Proximal lunate and lunate facet of radius) Two nonfunctional surfaces (Proximal and distal lunate)
Grade3	Three nonfunctional surfaces (All except capitate surface)
Grade4	All four articular surfaces are nonfunctional

In addition to radiographic and perfusion-based classifications, ***Bain and Begg*** proposed an arthroscopic grading system (Table 1) to assess the functional integrity of articular surfaces surrounding the lunate. This classification evaluates the progressive degeneration of intermediate wrist column [5], with initial arthritic changes affecting the proximal lunate surface, followed by secondary involvement of the lunate facet of the radius. As the disease advances, degeneration extends to the distal lunate articular surface and eventually the proximal capitate surface [6]. While this arthroscopic grading system provides a practical treatment guide based on the extent of arthritis, advanced imaging modalities such as MRI and computed tomography (CT) can also be utilized to assess the integrity of articular surfaces [4, 7].

**Fig. 1:**
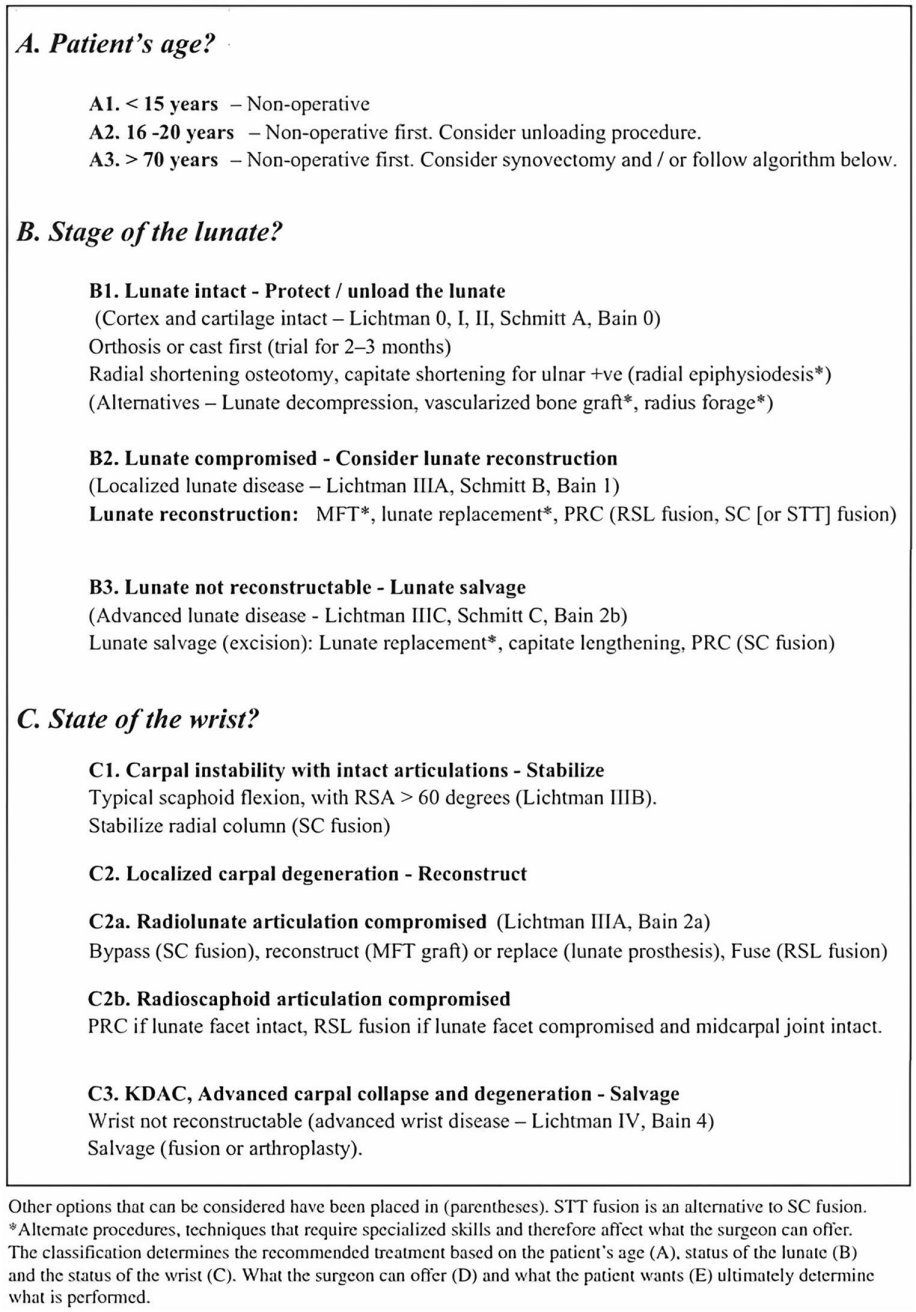
The Bain and Lichtman algorithm for management of KD of the wrist [8]

The surgical management of KD is broadly classified into five categories: decompression, revascularization, unloading, reconstruction, and salvage procedures. However, relying solely on a single classification system often provides an incomplete picture, leaving ambiguities regarding the optimal treatment strategy at various disease stages. Recognizing these limitations, a recently developed treatment algorithm integrates three key classification systems [2, 4]: the traditional osseous staging (***Lichtman***), the perfusion-based system (***Schmitt***), and the articular cartilage status (***Bain***). This comprehensive approach accounts for the structural integrity of the lunate, its vascular viability, and the condition of surrounding joint surfaces (Fig. 1). ***Lichtman and Bain*** further refined this algorithm by incorporating critical patient- and surgeon-related factors, including (A) patient age, (B) lunate stage, (C) overall wrist condition, (D) surgeon expertise, and (E) patient preferences. This multidimensional framework aims to guide decision-making in a more individualized and evidence-based manner, ultimately optimizing patient outcomes in KD management [8, 9].

The lunate may develop post-collapse bony protuberances (PCBPs), which resemble bossing or exostosis on the dorsal and volar surfaces. Patients with late-stage KD, especially those from Stage III onward, frequently have persistent pain and noticeably weaker grips, which makes it difficult for them to perform daily and work-related tasks. Patients with advanced KD are usually treated with salvage procedures [10]. One targeted carpal fusion technique that has shown promise in treating patients with significant joint degeneration and incongruence is scaphocapitate fusion [11]. Our study evaluated the clinical and radiological outcomes of scaphocapitate fusion (SCF) in KD stage IIIA and IIIB. It also examined the impact of lunate management—complete excision (Lunarectomy), or preservation—on surgical results. This will help to determine the most effective approach for enhancing patient function and relieving symptoms in patients with advanced KD.

## Materials and methods

**Fig. 2:**
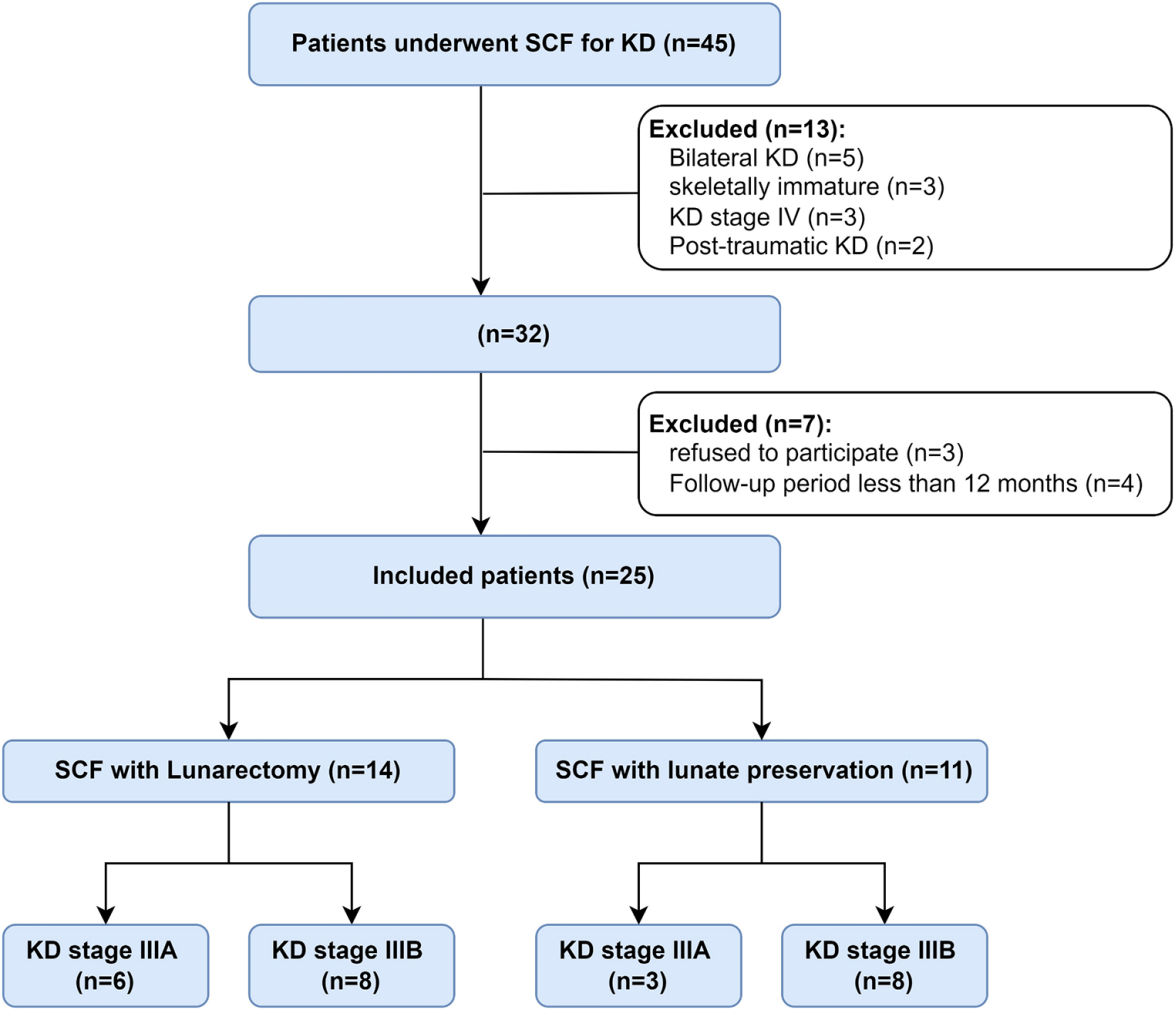
The flowchart of patients’ selection process

This study has been approved by the institutional review board of the authors. It involved 25 patients with stage III KD who underwent SCF treatment and were followed up between December 2019 and August 2024. ***The inclusion criteria*** were: (1) A diagnosis of KD Stage IIIA or IIIB based on the Lichtman classification, (2) Skeletally mature patients over the age of 18. ***Exclusion criteria*** were: (1) Bilateral KD, (2) Skeletally immature patients, (3) KD stage I, II, or IV, (4) Post-traumatic KD (following peri-lunate dislocation injuries), and (5) Follow-up period of less than 12 months. All available surgical alternatives such as capitate shortening or proximal row carpectomy (PRC) were explained to the patients who were included in the study. Patients with prior surgical interventions for KD, such as a radial shortening procedure, were not excluded from this study. Every patient received information regarding the potential benefits and drawbacks of each procedure. All patients provided written informed consent to participate in the study. The flowchart for the patient selection process is demonstrated in Fig. 2.

In this retrospective analysis, 25 patients with KD IIIA (*n* = 9) and IIIB (*n* = 16) were included. Patients’ detailed demographics are presented in Table 2. Standard radiographs (anteroposterior (AP) and lateral views) were obtained for KD staging and diagnosis confirmation. Additionally, MRI or CT scans were performed to evaluate the degree of lunate collapse, carpal alignment, and joint degeneration. The Stahl’s lunate height index (LHI), Youm’s carpal height ratio (CHR), and modified capitate height ratio (modified-CHR), also referred to as the Nattrass index, radio-scaphoid angle (RSA), and modified carpal-ulnar distance ratio (CUDR), were reported using pre- and post-fusion radiographs. The LHI is the ratio of the lunate length in the AP view and its AP diameter in the lateral view [12]. The CHR is the ratio of the total carpal height (TCH) to the length of the third metacarpal (MCB) on AP radiographs [13, 14]. The RSA is the angle between the line tangent to the volar aspect of the scaphoid and the longitudinal axis of the distal radius [15]. The modified-CHR is the ratio of the distance between the base of the third MCB and the distal articular surface of the radius to the longitudinal length of the capitate [16]. The modified-CUDR is the ratio of the distance between the center of the capitate head and the longitudinal axis of the ulna to the longitudinal length of the capitate [17].

**Table 2:** Patients’ demographics

Variable	Total (*n* = 25)	Stage IIIA (*n* = 9)	Stage IIIB (*n* = 16)
Age (Mean ± SD) (y)	30.2 ± 5.9 (20–38)	31.7 ± 6.8 (20–37)	29.3 ± 5.4 (22–38)
Gender (n, %)	Male (*n* = 17; 68%) Female (*n* = 8; 32%)	Male (*n* = 4; 44.4%) Female (*n* = 5; 55.5%)	Male (*n* = 13; 81.25%) Female (*n* = 3; 18.75%)
Side (n, %)	Right (*n* = 17; 68%) Left (*n* = 8; 32%)	Right (*n* = 5; 55.5%) Left (*n* = 4; 44.4%)	Right (*n* = 12; 75%) Left (*n* = 4; 25%)
Hand dominance (n, %)	D (*n* = 19; 76%) ND (*n* = 6; 24%)	D (*n* = 6; 66.6%) ND (*n* = 3; 33.3%)	D (*n* = 12; 75%) ND (*n* = 4; 25%)
Occupation (n, %)	ST (*n* = 7; 28%); HW (*n* = 6; 24%); MW (*n* = 12; 48%)	ST (*n* = 2; 22.2%); HW (*n* = 4; 44.4%); MW (*n* = 3; 33.3%)	ST (*n* = 5; 31.25%); HW (*n* = 2; 12.5%); MW (*n* = 9; 56.25%)

n: Number, D: Dominant, ND: Non dominant, ST: Student, HW: Housewife, MW: Manual worker

**Fig. 3:**
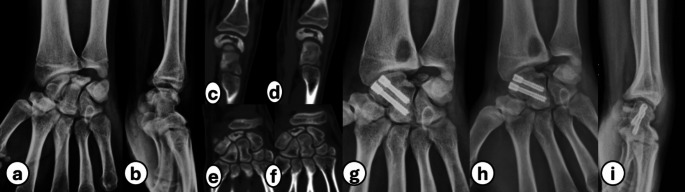
(**a**, **b**): AP and lateral wrist radiographs demonstrate stage IIIB KD with lunate collapse and fracture. (**c-f**): Sagittal and coronal CT slices show collapsed lunate with fragmentation. (**g**, **h**, **i**): AP, scaphoid, and lateral wrist radiographs after 7 months of follow-up reveal lunarectomy and complete healing of the scaphocapitate fusion site with evidence of bone graft harvesting site at the distal radius

**Fig. 4:**
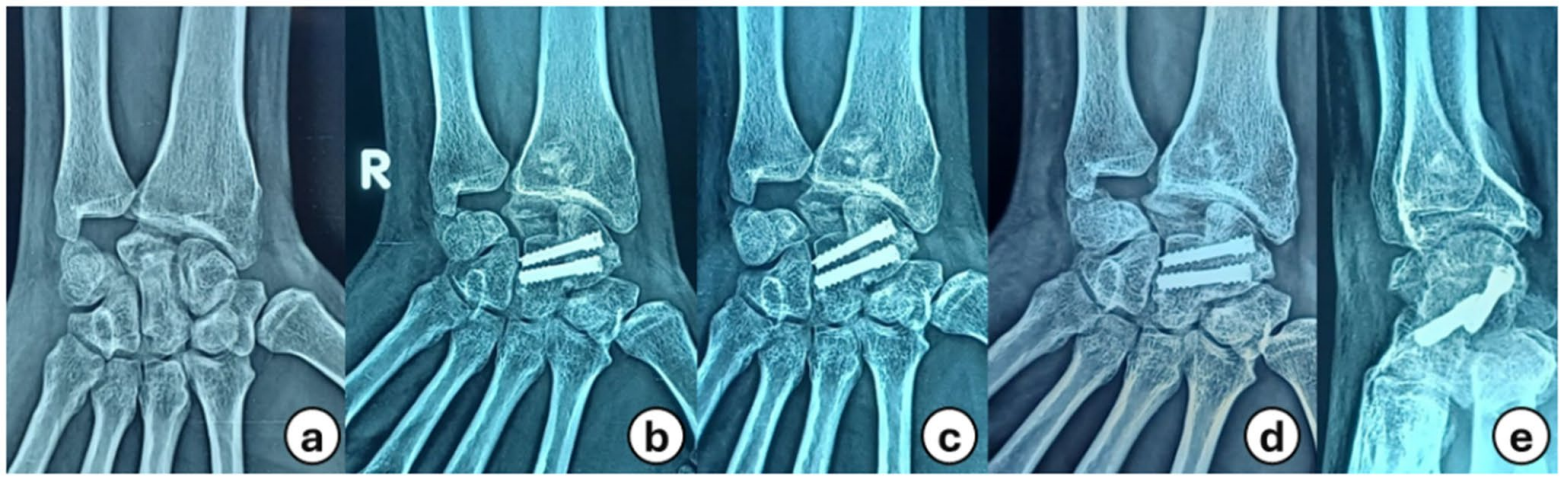
**a**: Preoperative AP radiograph on the right wrist showing stage III KD. (**b**, **c**): Scaphoid and AP views after 5 months show healed SCF site with lunate preservation. (**d**, **e**): Last AP and lateral wrist follow-up radiographs (35 months) with complete consolidation of the SCF site

**Fig. 5:**
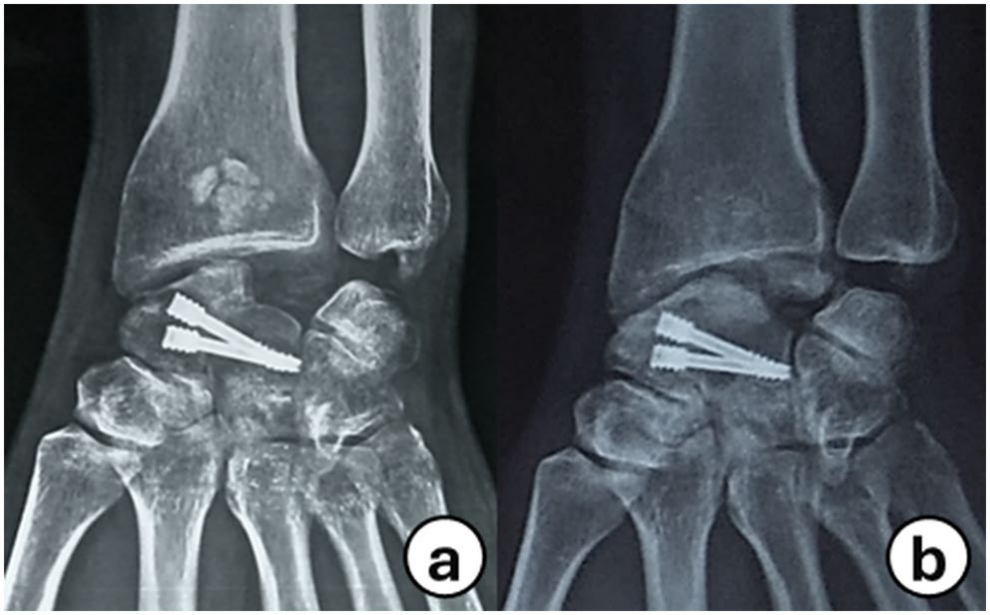
**a**: Postoperative AP wrist radiograph (4 months) for a 25-year-old male underwent SCF with Lunarectomy, and the excised lunate was implanted at the distal radius harvesting site. **b**: Final radiograph shows healing of the SCF site along with uptake of the lunate into the distal radius with full consolidation. Evidence of calcification at the site of excised lunate is noted

Pre- and postoperative clinical evaluations were performed for all patients regarding the pain level (measured by the Visual Analog Scale (VAS) [18]), active wrist range of motion (ROMs), and hand grip strength in kg using the Baseline ^®^ hydraulic hand dynamometer (Fabrication Enterprises, Elmsford, NY, U.S.A.), wrist function (assessed using the Modified Mayo Wrist Score (MMWS) [19], and the Disabilities of the Arm, Shoulder and Hand (DASH) [20]). Two independent orthopedic surgeons with at least five years of experience completed all clinical and radiological evaluations. Tables 3 and 4, and 5 demonstrate all preoperative and postoperative clinical and radiological findings (Figs. 3, 4 and 5).

**Table 3:** Preoperative and postoperative clinical and radiological results of all patients

Parameter	Preoperative	Postoperative	*P*-value
Clinical results			
Flexion (°)	52.6 ± 11.2 (30–65)	58.8 ± 6.5 (40–65)	0.051
Extension (°)	48 ± 90.68 (30–60)	61 ± 10.1 (40–75)	< 0.05*
Ulnar deviation (°)	24.3 ± 6.51 (15–35)	27 ± 4.5 (20–35)	0.14
Radial deviation (°)	11.2 ± 2.54 (8–15)	12.3 ± 2.01 (10–15)	0.144
Grip strength (kg)	17.3 ± 4.74 (6–24)	26.68 ± 3.3 (22–32)	< 0.05*
Grip strength ratio (%)	59.6 ± 16.2 (20–80)	90 ± 90.1 (75–100)	< 0.05*
VAS numerical score	4.84 ± 1.57 (2–8)	0.64 ± 0.9 (0–3)	< 0.05*
DASH score	48.36 ± 12.81 (28–74)	12.8 ± 3.61 (9–22)	< 0.05*
MMW score	51 ± 17.44 (15–70)	84.6 ± 7.06 (65–90)	< 0.05*
Radiological results			
RSA (°)	62.68 ± 10.27 (48–78)	45.88 ± 7.35 (38–65)	< 0.05*
CHR	0.41 ± 0.04 (0.32–0.46)	0.41 ± 0.08 (0.31–0.76)	0.19
LHI	0.43 ± 0.02 (0.4–0.48)	0.41 ± 0.02 (0.39–0.46)	0.33
Revised CHR	1.424 ± 0.13 (1.25–1.81)	1.33 ± 0.09 (1.15–1.54)	0.003*
Modified CUDR	0.83 ± 0.03 (0.72–0.85)	0.8 ± 0.06 (0.54–0.85)	0.008*

VAS: Visual analogue score, DASH score: Disabilities of Arm, Shoulder, and Hand score, MMW score: Modified Mayo Wrist score, RSA: Radio-scaphoid angle, CHR: Carpal height ratio, LHI: Lunate height index, CUDR: Carpal ulnar distance ratio, (°): Degrees, *: Statistically significant

**Table 4:** Influence of lunate management and KD stage on clinical results

Parameter	Lunate bone management			KD stage		
	Lunarectomy (*n* = 14)	Preservation (*n* = 11)	*P*-value	Stage IIIA (*n* = 9)	Stage IIIB (*n* = 16)	*P*-value
Flexion (°)	61.7 ± 3.1 (55–65)	55 ± 7.7 (40–65)	0.017*	60 ± 8.2 (40–65)	58.12 ± 5.4 (45–65)	0.19
Extension (°)	66.4 ± 7.1 (55–75)	54 ± 9.1 (40–75)	0.002*	57.7 ± 10 (40–75)	62.81 ± 9.9 (50–75)	0.33
Total ROM	128.2 ± 7.9 (115–145)	109.09 ± 16.8 (80–145)	0.001*	117.7 ± 16.6 (80–135)	120.9 ± 15.6 (90–145)	0.81
Total ROM (%)	91.2 ± 3.9 (85–98)	88 ± 7 (74–95)	0.32	91 ± 3.4 (86–98)	89.1 ± 6.5 (74–98)	0.53
Ulnar deviation (°)	26.8 ± 4.9 (20–32)	27 ± 4 (20–35)	0.82	24.4 ± 4.7 (20–32)	28.3 ± 3.7 (20–35)	0.06
Radial deviation (°)	12.2 ± 2.1 (10–15)	12.27 ± 1.9 (10–15)	0.96	12.7 ± 1.7 (10–15)	12 ± 2.1 (10–15)	0.3
Grip strength (kg)	27.9 ± 3.1 (22–32)	25 ± 2.9 (22–32)	0.07	27 ± 3.3 (22–30)	26.5 ± 3.4 (23–32)	0.81
Strength ratio (%)	95.3 ± 6 (80–100)	83.1 ± 7.5 (75–100)	0.002*	91.1 ± 7.4 (80–100)	89.37 ± 9.9 (75–100)	0.95
VAS score	0.21 ± 0.0.4 (0–1)	1.18 ± 1.1 (0–3)	0.037*	0.44 ± 0.7 (0–2)	0.75 ± 1 (0–3)	0.61
DASH score	11.57 ± 1.9 (9–16)	14.2 ± 7.3 (38–65)	0.18	12.1 ± 3.5 (10–21)	13.12 ± 3.6 (0–3)	0.33
MMW score	88.57 ± 3.6 (80–90)	79.54 ± 7.2 (65–90)	0.003*	85.5 ± 7.2 (70–90)	84 ± 7.1 (65–90)	0.58
Operative time (min)	84.6 ± 10.2 (70–105)	81.3 ± 15.5 (65–105)	0.39	80 ± 8.6 (70–95)	85 ± 14.3 (65–105)	0.45
Follow-up duration (m)	27.5 ± 3.3 (24–36)	29.18 ± 5 (24–40)	0.52	29.1 ± 3.6 (25–36)	27.8 ± 4.4 (24–40)	0.21
Healing time (m)	15.69 ± 2.5 (12–20)	15.5 ± 2.7 (12–20)	0.82	15.3 ± 2.7 (12–20)	15.7 ± 2.5 (12–20)	0.79

KD: Kienbock disease, ROM: Range of motion, VAS: Visual analogue score, DASH score: Disabilities of Arm, Shoulder, and Hand score, MMW score: Modified Mayo Wrist score, min: Minutes, m: Month, (°): Degrees, *: Statistically significant

**Table 5:** Influence of lunate management and KD stage on radiological results

Parameter	Lunate bone management			KD stage		
	Lunarectomy (*n* = 14)	Preservation (*n* = 11)	*P*-value	Stage IIIA (*n* = 9)	Stage IIIB (*n* = 16)	*P*-value
RSA (°)	45.7 ± 7.6 (38–60)	46 ± 7.3 (38–65)	0.96	44.2 ± 4.2 (38–50)	46.8 ± 8.6 (38–65)	0.77
CHR	0.42 ± 0.1 (0.31–0.76)	0.39 ± 0.05 (0.31–0.45)	0.54	0.44 ± 0.12 (0.31–0.76)	0.39 ± 0.04 (0.31–0.45)	0.37
Revised CHR	1.32 ± 0.09 (1.15–1.45)	1.35 ± 0.09 (1.2–1.54)	0.5	1.4 ± 0.06 (1.33–1.54)	1.29 ± 0.08 (1.15–1.42)	0.02*
Modified CUDR	0.81 ± 0.02 (0.76–0.85)	0.78 ± 0.08 (0.54–0.85)	0.65	0.79 ± 0.09 (0.54–0.85)	0.8 ± 0.03 (0.75–0.85)	0.75
LHI	- (*n* = 14)	0.41 ± 0.02 (0.39–0.46) (*n* = 11)		0.414 ± 0.023 (0.41–0.44) (*n* = 3)	0.416 ± 0.026 (0.39–0.46) (*n* = 8)	

KD: Kienbock disease, RSA: Radio-scaphoid angle, CHR: Carpal height ratio, LHI: Lunate height index, CUDR: Carpal ulnar distance ratio, (°): Degrees, *: Statistically significant

### Surgical procedure

The patient was placed supine under general or regional anesthesia, and the arm was positioned on a sterile, radiolucent operating table. The arm was elevated and exsanguinated before inflating the pneumatic tourniquet. Prophylactic intravenous antibiotics were administered prior to tourniquet inflation. A dorsal wrist approach was used in every case [21]. A longitudinal incision, measuring eight to ten centimeters (cm), was made across the dorsum of the wrist, ulnar to Lister’s tubercle, in line with the axis of the third MCB bone. The subcutaneous tissue was dissected off the extensor retinaculum, creating thick flaps to protect the dorsal cutaneous nerves within these flaps.

After opening their sheaths, the tendons of the third and fourth compartments were identified and separated. A 2 cm portion of the posterior interosseous nerve (PIN) was excised, and the proximal end was cauterized to prevent the formation of a painful terminal neuroma. The fascial sheath of the third compartment was incised, and the extensor pollicis longus (EPL) tendon was completely released from its compartment. Subsequently, the tendons of both the second and third compartments were retracted laterally. A ligament-preserving capsulotomy was performed by dividing the dorsal wrist capsule along the dorsal intercarpal (IC) and radiocarpal (RC) ligaments, creating a Z-shaped capsular incision. When repaired, this technique is expected to minimize capsular contracture and improve postoperative ROM [22]. A radially based capsular flap was elevated off the triquetrum and reflected to expose the carpal bones.

The scaphoid, lunate, and capitate bones were identified. The radial scaphoid fossa was carefully inspected. In 14 patients (56%) a lunarectomy was done; in 11 patients (44%), the lunate was preserved. Lunarectomy was favored in patients with significant collapse with PCBPs and KD-IIIB. The lunarectomy was facilitated by maximal wrist flexion. Care was taken not to injure the volar IC ligaments. Post-lunarectomy, the superior articular surface of the capitate was completely visualized. As a joystick, a 2 mm (mm)-Kirschner wire (K-wire) was advanced into the scaphoid to manipulate the scaphoid bone and correct its rotation, particularly in KD IIIB cases. After that, the SC-joint (SCJ) was prepared. A high-speed burr, bone curettes, small bone rongeurs, and an 11 mm scalpel were used to denude and remove the articular cartilage and underlying subchondral bone. It was ascertained that the scaphoid and capitate volar articular cartilages were preserved. Furthermore, the volar radioscaphocapitate (RSC) ligament remained intact. Following the preparation of the SCJ, cancellous bone graft was employed and impacted inside the joint. With a substantial amount, three patients (12%) received the grafting from both the iliac crest and the distal radius, while 22 patients (88%) received it only from the radius. To prevent future stress fractures of the distal radius post-harvesting, the excised lunate was prepared (covering cartilage removal) and used as a bone void to occupy the distal radius defect (*n* = 9; 64.2% of lunarectomy-patients).

The scaphoid overhung the capitate after the joystick K-wire adjusted the scaphoid’s flexion and rotation. To allow for the advancement of the two guidewires across the SC articulation, an additional incision was made over the anatomical snuff box. In five patients (20%), a partial radial styloidectomy was performed. Extreme caution was used to avoid damaging the superficial radial nerve’s branches or the radial artery. Following a fluoroscopic examination to confirm the guidewire placement and scaphoid orientation, two appropriately sized headless compression screws (HCS) were inserted to stabilize the SC fusion. Screw advancement is facilitated by ulnar deviation. After removing the joystick wire, the wrist ROMs were inspected. After assuring hemostasis and deflating the tourniquet, the wound was irrigated. In every case, the Z-shaped, ligament-sparing capsulotomy was repaired. The extensor tendons were repositioned, and the edges of the extensor retinaculum were approximated to prevent bowstringing. Additionally, in all patients, the EPL tendon was transposed dorsally to the retinaculum to minimize the risk of postoperative tendinitis and stenosis. The wound was then closed in layers, and after applying a sterile dressing, the arm was immobilized in a neutral or slightly extended position with a short-arm volar splint.

Postoperative care involved encouraging limb elevation during the day to prevent edema. Active movements of the fingers, elbow, and shoulder were encouraged starting from the first postoperative day. At two weeks post-surgery, the stitches were removed, and the splint was replaced with a removable wrist orthosis, which was used until radiographic evidence of healing was observed. A hand physiotherapist with specialized training in hand rehabilitation also began to supervise the rehabilitation program, which prioritized ROM and strengthening exercises. After radiographic healing was confirmed, the patients were permitted to resume their heavy occupational activities.

### Postoperative follow-up

Patients were evaluated every four weeks to assess fusion union after cast removal. The union was evaluated with standard AP and lateral wrist radiographs through bridging trabeculae across the fusion site, no SCJ gap, no peri-joint lucency, and well-positioned hardware. SCF was not regarded as united until it was verified by two specialized hand surgeons and a musculoskeletal radiologist. Furthermore, carpal alignment and wrist congruency were monitored on follow-up radiographs, and any related complications—such as arthritis, nonunion, or hardware positional alteration—were documented. When a patient reached six months postoperatively and there was no discernible radiographic healing (non-obvious bridging trabeculae, SCJ gap, peri-joint lucency, or altered hardware position on follow-up radiographs), the SCF was considered non-united.

### Statistical analysis

Statistical analysis was conducted using. IBM SPSS Statistics for Windows, Version 29.0.2.0 (IBM Corp. Released 2023, Armonk, NY). Descriptive statistics, including frequency, percentage, mean, and standard deviation, were calculated to summarize the data. Normality was assessed using the Kolmogorov-Smirnov test. For normally distributed data, the independent samples t-test was used to compare postoperative outcomes between the lunarectomy and lunate-preservation groups, while the Mann–Whitney U test was applied for non-normally distributed data. Categorical variables were analyzed using the chi-square test. Additionally, differences between preoperative and postoperative clinical and radiological findings were assessed across all patients using the appropriate tests based on data distribution. A significant level of *P* < 0.05 was set for all statistical tests.

## Results

According to the Lichtman classification system, nine patients (36%) were classified as stage IIIA, while sixteen patients (64%) were categorized as stage IIIB. Manual laborers represented 48% of the patient population. The right wrist and the dominant wrist were most frequently impacted, affecting 68% and 76% of the patients, respectively. Eleven patients (44%) had their lunate preserved, and fourteen patients (56%) had a lunarectomy during the SCF procedure. Three patients (12%) underwent SCF following radial shortening, a joint leveling procedure. Across all patients, the mean operative time was 83.2 ± 12.6 min (range: 65–105). Lunate excision or preservation did not significantly affect the operative time (*P* = 0.39). Patients completed a mean follow-up period of 28.28 ± 4.17 months (range: 24–40).

Clinically, all patients demonstrated a significant improvement in the total (flexion-extension) ROM (*P* = 0.0006) and total ROM percentage (*P* < 0.05) following surgery. Likewise, there was a significant improvement (*P* < 0.05) in the grip strength ratio, VAS scale, DASH, and MMW scores. In the same context, KD IIIA and IIIB did not significantly differ in their SCF’s clinical outcomes; nonetheless, KD IIIA patients had marginally better clinical outcomes. On the other hand, compared to patients with lunate preservation, SCF with a lunarectomy demonstrated significant improvements in terms of total ROM (*P* = 0.001), grip strength ratio (*P* = 0.0023), and MMW score (*P* = 0.0033). Furthermore, the pain score was relevantly lower (*P* = 0.037). Comprehensive functional outcomes for all patients are shown in Tables 3 and 4.

In 23 patients (92%), the SCF site was successfully consolidated in an average of 15.6 ± 2.5 weeks (range: 12–20). The average radio-scaphoid angle decreased significantly to 45.8 ± 7.3° (*P* < 0.05). Furthermore, there was a decline in the mean postoperative modified-CHR and modified-CUDR (*P* = 0.003 and 0.008, respectively). The postoperative radiographic calibrations after SCF with either lunate preservation or lunarectomy did not show any significant differences (*P* > 0.05). Tables 3 and 5 present the radiographic calibrations in detail.

**Fig. 6:**
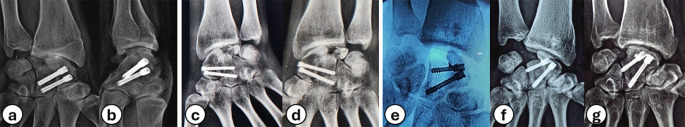
Complications following SCF among study’s participants. (**a**, **b**): AP and lateral wrist radiographs demonstrate radio-scaphoid osteoarthritis with healed SCF. (**c**, ** d**): Scaphoid and AP wrist radiographs of SCF nonunion with lunate bone remnants. (**e**, **f**): Intraoperative image and postoperative wrist radiographs show a 2 mm fragment of the drill bit broken during reaming and left in the scaphoid. **g**: complete radiographic healing of the SCJ with ulnar carpal translation on final radiographs

Out of the patient cohort, two individuals (8%) experienced radiological nonunion at the fusion site. One patient, classified as stage IIIA KD, underwent a lunarectomy (Fig. 6c, d), while in the other, with stage IIIB KD, the lunate was preserved. Both patients reported manageable mechanical wrist pain, with a postoperative VAS score of 1, and declined further surgical intervention. The patient who underwent lunarectomy showed excellent results, with a MMWS of 90, total ROM of 120°, and grip strength ratio at 95%. The patient with lunate preservation exhibited fair outcomes, with an MMWS of 65, total ROM of 90°, and grip strength at 75%. A superficial wound infection occurred in one patient (4%) and was effectively treated with oral antibiotics. Two patients (8%) developed RS arthritis, which did not result in clinical symptoms and did not require any intervention. Intraoperatively, in one case, a 2 mm fragment of the drill bit broke during reaming and was intentionally left in the scaphoid to prevent additional injury (Fig. 6e). The SCJ demonstrated complete healing with no clinical symptoms, despite radiographic evidence of ulnar carpal translation on final radiographs (Fig. 6g). Notably, no iatrogenic or stress fractures were observed at the graft harvesting site in the distal radius, and there were no hardware-related complications.

## Discussion

The progressive collapse of the central column primarily impairs the mechanics and function of the wrist as Kienböck’s disease advances. This is followed by degeneration and instability around the peri-lunate region, disrupting the proximal row between the central and radial columns. Over time, this process culminates in radiocarpal joint degeneration, which leads to advanced collapse (KD-AC). The resultant degeneration and instability often disrupt the delicate balance of the SL ligament. Consequently, the capitate becomes wedged into the SL interval as the central column height decreases (nutcracker effect). The trapezium forces the scaphoid into flexion, causing the lunate to extend, which results in dorsal intercalated segment instability (DISI) [1]. The SCF concept aims to bypass the central column, thereby reducing joint forces at the RL and Lunocapitate (LC) joints and decreasing tension on the SL ligament, ultimately preventing further carpal collapse. This allows for painless wrist mobilization through a stabilized radial column [23].

Our study demonstrated that SCF, as an effective limited IC fusion procedure, significantly improved the mean grip strength ratio from 59.6 ± 16.2% to 90 ± 90.1%. Similarly, ***Hegazy et al.*** [24]. and ***Luegmair and Saffar*** [25] reported mean postoperative grip strengths of 79% and 64% of the contralateral side, respectively. Additionally, significant improvement in mean grip strength of the operated side was widely reported in literature [10, 11, 17, 26–29]. The superior grip strength outcomes in our study may be partly due to the pre-injury occupational status of a significant portion of patients (48%; manual workers) and the stronger baseline grip strength in the dominant hand (76%), especially among right-handed individuals (68%). The natural strength advantage of the dominant hand likely contributed to the improved postoperative results. Although earlier investigations have highlighted the need to adjust grip strength measurements for this asymmetry [30, 31], our analysis did not make such corrections. This lack of adjustment could explain the higher postoperative grip strength observed and underscores the importance of accounting for hand dominance in future research for more accurate interpretations.

The impact of SCF on ROM varied across studies. Many reported a decrease in mean flexion ROM [25–27], ranging from a minimum of 5° [25] to a maximum of 16° [26], and in extension ROM [25–27], ranging from a minimum of 9° [26] to a maximum of 14° [25]. In contrast, ***Özdemir et al.*** [29] and ***Charre et al.*** [28] observed an increase in mean flexion and extension ROMs by less than 8°. ***Meena et al.*** [10] reported a significant increase in mean flexion and extension ROMs by more than 10° (*P* < 0.05). Our study showed an improvement in mean flexion and extension by 5° and 13°, respectively (*P* = 0.051 and *P* = 0.0001). This increased ROM might be attributed to the patients’ category contributed to this investigation, which excluded stage IV KD and included a considerable percentage of early KD stage (stage IIIA), representing one-third (33.3%) of the patients. Additionally, preoperative ROM measurements might have been significantly underestimated due to patient pain. All studies, including ours, indicated that the final ROM was satisfactory and did not interfere with occupational or daily tasks.

In this study, as in most earlier reports, we used the traditional dorsal wrist approach, making a longitudinal incision ulnar to the Lister’s tubercle along the third MCB’s axis. While this was the primary incision, we also made a smaller lateral incision over the anatomical snuffbox for screws insertion. This separate incision was chosen to avoid injury to the radial artery or superficial radial nerve branches, instead of using a percutaneous approach. In contrast, ***Hegazy et al.*** [24] employed an S-shaped incision extending from the second MCB’s base to nearly 2 cm proximal to lister’s tubercle, for both access and screw insertion under direct visualization, eliminating the need for a second incision, as no lunarectomy was performed. Similarly, ***Meena et al.*** opted for a more radial incision without exposing the lunate, as lunarectomy was also not part of their procedure [10].

Earlier studies have explored various capsulotomy designs during SCF procedures, each with the goal of optimizing outcomes in patients with KD. ***Eid et al.*** [32]. utilized a longitudinal capsulotomy over the SC interval and observed an improvement in grip strength (63%), although many patients (78.9%) continued to experience persistent pain. Similarly, ***Hegazy et al.*** [24]., using the same design, reported no significant improvement in flexion-extension ROM, but did find relevant improvements in grip strength ratio and pain levels. ***Meena et al.*** [10], employing a longitudinal capsulotomy without exposing the lunate, reported significant gains in both ROM and grip strength ratio. In contrast, ***Charre et al.*** [28] used a T-shaped capsulotomy and encountered less favorable results, including wrist stiffness in 50% of patients, loss in hand strength in 22.2%, and chronic pain in 27.7% of cases. ***Park et al.*** [17], also employing a T-shaped capsulotomy, reported a significant improvement in grip strength, though with a slight decline in ROM. Our study, which utilized a Z-shaped capsulotomy, demonstrated significant improvements in extension ROM, grip strength ratio, and pain levels. While these findings highlight some associations between capsulotomy design and clinical outcomes, it remains challenging to attribute these outcomes solely to the capsulotomy design as an independent variable. Other important factors must be considered to fully assess postoperative results, including the patient’s preoperative clinical condition, the management of the lunate bone during surgery, and the subsequent postoperative rehabilitation process. These elements are crucial in influencing the overall clinical outcomes and may contribute to variability in results.

In wrist surgeries, different capsulotomy techniques have been described, including longitudinal, transverse, oblique, T-shaped, L-shaped, Z-shaped, distal-based U-shaped [33], and proximal-based U-shaped designs [34]. ***Berger et al.*** introduced the V-shaped fiber-splitting arthrotomy, which provides excellent visualization of the RC and midcarpal joints [35]. However, ***Hagert et al.*** identified several potential drawbacks, such as damage to the dorsal RC and IC ligaments, disruption of PIN innervation, and risk to dorsal wrist vascularity [36]. These factors are critical for postoperative wrist proprioception and rehabilitation. In accordance with early literature, a 2 cm distal branch of the PIN was excised in all study participants. That resulted in a significant pain reduction (*P* < 0.05), with a mean postoperative VAS score of 0.64 ± 0.9 (range 0–3). Capsulotomy design plays a vital role in preserving dorsal joint vascularization and innervation [37], especially when it involves a transverse limb near the distal radius. Such limb poses a significant risk of damaging the vascular supply to the dorsal capsule, potentially leading to healing problems and capsular scarring. To address these concerns, ***Athlani et al.*** proposed a U-shaped capsulotomy with a proximal base, which preserves neurovascular integrity, provides versatile joint exposure, and reduces tension during capsular repair, thereby minimizing the risk of capsular scarring and wrist stiffness [34].

In our study, a 92% union rate for SCF was achieved using two HCS and a DR bone graft in all participants. While most studies employed two screws for fusion [10, 17, 23, 24, 27, 28, 38–40], ***Goyal et al.*** reported a 100% union rate even when using a single screw in some cases [26].

In the same context, different studies reported complete SCF union with the exclusive use of HCS [26, 40, 41]. However, different implants were also utilized in other investigations. ***Park et al.*** [17] reported the use of HCS, K-wires, or a combination of both, while ***Zakzouk*** [42] and ***Hasan et al.*** [39]. used either K-wires or HCS. ***Rhee et al.*** [27] employed various fixation devices, including HCS (63%), staples (26%), HCS with K-wires (7%), and HCS with staples (4%), while ***Eid et al.*** [32] used K-wires in 84% of patients and staples in 16%. Despite the differences in fixation methods, these studies all reported 100% union rates. However, complications occurred in some cases, such as K-wire removal in 5.1% of patients in ***Park et al.’s*** study [17] and staple removal in 3.7% of patients in ***Rhee et al.’s*** study [27].

Union rates of 90.6% and 92.5% were demonstrated by ***Hegazy et al.*** [24] and ***Meena et al.*** [10]. with the use of HCS. While ***Pisano et al.*** [23]. and ***Szalay et al.*** [38] observed relatively lower union rates of 88% and 80%, respectively, using K-wires, screws, or staples for fixation. ***Charre et al.*** [28]. reported a 94.4% union rate with various implants, including K-wires (61.1%), staples (33.3%), and HCS (5.5%), and ***Luegmair and Saffar*** [25] demonstrated a 93.3% union rate with either dorsal plates or staples.

In our study, the use of headless compression screws for fixation enabled early range of motion (ROM) in all patients, with a brief immobilization period involving a splint for only two weeks post-surgery, followed by a removable orthosis. ***Kayaokay et al.*** [41] reported a similar postoperative protocol, utilizing a splint for three weeks, followed by a removable splint for an additional six weeks. In contrast, numerous reports by ***Rhee et al.*** [27], ***Charre et al.*** [28]., ***Eid et al.*** [32], and ***Luegmair and Saffar*** [25], who used K-wires and staples for fixation, recommended a more extended period of immobilization. These studies favored delaying wrist motion for at least eight weeks, during which patients were kept in a non-removable splint until K-wires were removed or radiographic confirmation of healing was achieved.

The distal radius bone graft was the most used graft for SCF, with several studies reporting full union using this graft [26, 32, 39, 40, 42]. However, slightly lower union rates were seen in some studies, such as ***Hegazy et al.*** (90.6%) [24], ***Meena et al.***. (92.5%) [10], and ***Luegmair et al.*** (93.3%) [25]. ***Park et al.*** reported a 100% union rate using IC grafts [17]. Other studies, including those by ***Pisano et al.*** [23] and ***Charee et al.*** [28], reported union rates of 88% and 94.4%, respectively, when using either DR or IC grafts. ***Rhee et al.*** [27] reported 100% union using DR grafts (55.5%), IC grafts (37%), and DR combined with demineralized bone matrix (7.4%). Overall, graft usage-whether DR or IC-consistently resulted in high union rates across studies, regardless of the fixation method used. Both graft types were associated with successful outcomes, and fixation methods, including K-wires, HCS, and staples, were similarly effective. However, complications such as infections and stiffness were more commonly linked to K-wires and staples. Although graft usage is crucial for achieving successful union, the specific type of graft or fixation device alone does not seem to be the sole determinant of union rates, with other factors like patient condition and surgical technique playing significant roles.

One of the key aspects of this study was comparing the outcomes of different lunate bone management options (lunarectomy versus preservation). This comparison has not been extensively discussed in literature. Our study found that SCF with lunarectomy resulted in superior postoperative outcomes compared to SCF with lunate preservation. Specifically in terms of total ROM (*P* = 0.001), grip strength percentage (*P* = 0.0023), and MMW scores (*P* = 0.0033). Additionally, the pain level was significantly lower after lunarectomy (*P* = 0.037). These findings align with those of **Luegmair and Saffar** [25], who reported better outcomes in terms of flexion and pain level with lunarectomy compared to lunate preservation. However, the mean grip strength was nearly the same between the two groups. Detailed patient-reported outcomes are presented in Table 6.

**Table 6:** Results of previous studies of SCF in advanced stages of Kienbock disease

Study/year	*N* (M/F)	Age (y)	KD stage	Lunate management	Follow-up duration	Clinical findings	Radiological outcome	Complications
Current study	25 (17/8)	30.2 ± 5.9 (20–38)	IIIA, IIIB	Lunarectomy (*n* = 14), preservation (*n* = 11)	28.2 ± 4.1 m (24–40).	Flexion 58.8 ± 6.5 (40–65), Extension 61 ± 10.1 (40–75), Ulnar deviation 27 ± 4.5 (20–35), Radial deviation, Grip strength 26.68 ± 3.3 kg (22–32)12.3 ± 2.01 (10–15), Grip strength (%) 90 ± 90.1 (75–100), VAS 0.64 ± 0.9 (0–3), DASH 12.8 ± 3.61 (9–22), MMW 84.6 ± 7.06 (65–90)	RSA 45.88 ± 7.35 (38–65), CHR 0.41 ± 0.08 (0.31–0.76), LHI 0.41 ± 0.02 (0.39–0.46), modified CHR 1.33 ± 0.09 (1.15–1.54), Modified CUDR 0.8 ± 0.06 (0.54–0.85)	Nonunion (*n* = 2), superficial wound infection (*n* = 1), RS arthritis (*n* = 2)
Hegazy et al. [24], 2024	32 (19/13)	33 (19–48)	IIIB, IIIC	Preservation	45.6 m (33–56)	Total ROM (%) 49 (44–51), VAS score 1.3 (0-2.5), Grip strength (%)79 (74–85), MMWS 75 (70–80), Quick-DASH scores 21 (18–48)	RS angle 50 (46–60), LHI 0.42 (0.45–0.49), CHI 0.42 (0.40–0.48)	Nonunion (*n* = 3); underwent revision using an iliac crest BG graft, wound infections (*n* = 2), scar hypertrophy (*n* = 2), CRPS (*n* = 1), radial side wrist pain (*n* = 1), pericarpal arthritis (*n* = 8)
Park et al. [17], 2022	39 (18/21)	44 (22–73)	IIIA, IIIB, IV	Lunarectomy	40 m (12 m- 12y)	Flexion–extension arc 71.4 ± 13.6 (40–93), Radioulnar deviation arc 34.0 ± 8.5 (9–53), VAS 1.1 ± 1.1 (0–4), Grip strength 51.2 ± 20.6 lb. (10–102), MMWS 69.5 ± 9.3 (40–90), Quick-DASH 21.5 ± 10.4 (9.1–54.5)	NR	RS arthritis (*n* = 12) *Reoperations*: Removal of K-wires (*n* = 2)
Meena et al. [10], 2022	23 (9/14)	30 (19–47)	IIIA, IIIB	Preservation	8.1 y (7–10)	Flexion 45 ± 14, Extension 46 ± 12, Radial deviation 10 ± 8, Ulnar deviation 20 ± 9, DASH 28 (7–65), PRWE 31 (2–69), VAS 4 (0–7), Grip strength 32 ± 7 kg	Modified CHR 1.18 ± 0.37, Modified CUDR 0.66 ± 0.14, RS angle 47 ± 9, SL Angle 28 ± 12, Stahl Index 0.39 ± 0.1	Nonunion (*n* = 2)
Goyal et al. [26], 2020	11 (3/8)	24 (17–45)	IIIA, IIIB	Both performed (not specified)	18 m (18–26)	Flexion 35 ± 9.34 (15–50), Extension 36 ± 13.05 (20–60), Ulnar deviation 15 ± 5.22 (10–25), Radial deviation 12 ± 3.43 (10–20), VAS 2 ± 1 (1–4), Grip strength 26.09 ± 4.76 kg (18–34)	RS angle 48.3 ± 6.77 (40–60), SL angle 26.27 ± 5.9 (16–35), CHR 0.45 ± 0.02	No complications
Özdemir et al. [29], 2017	9 (3/6)	33.2 ± 11 (18–54)	IIIB	Lunarectomy	17.3 ± 4.6 m (12–24)	Extension 27.7 ± 4.4, flexion 40.5 ± 4.6, VAS 1.4 ± 0.5, Grip strength 71 ± 6.4	Modified CHR 1.39 ± 0.03, SL angle 55 ± 6.61	No complications
Charre et al. [28], 2017	17 (7/10)	36 (19–56)	IIIA, IIIB, IV	Lunarectomy	10.7 y (2.3–22)	Flexion 40 ± 14 (20–70), Extension 45 ± 15 (25–70), Ulnar tilt 30 ± 6 (20–40), Radial tilt 12 ± 3 (6–20), Grip strength 25.8 ± 8.4 kg (10–40), VAS 2.4 ± 2 (0–6)	RS angle 58 ± 11 (25–73), SC angle 72 ± 16 (32–95), CHR 0.45 ± 0.04 (0.4–0.54)	CRPS (*n* = 2), Superficial infections (*n* = 2), nonunion (*n* = 1), RS Arthritis (*n* = 2)
Rhee et al. [27], 2015	27 (17/11)	41 (15–66)	III, IV	Preservation (*n* = 11) Subtotal lunarectomy (*n* = 12), Partial lunarectomy (*n* = 4)	60 m (12 m-16y)	Flexion 30 ± 17, Extension 36 ± 13, Ulnar deviation 23 ± 9, radial deviation 11 ± 8, Grip strength 24 ± 14 kg	Modified CUDR 0.80 ± 0.14, modified CHR 1.34 ± 0.11, RS angle 43 ± 9	Persistent pain (*n* = 5), CRPS (*n* = 2), IC arthritis (*n* = 11) *Reoperations*: Radial styloidectomy (*n* = 1), Partial wrist denervation and ulnar styloidectomy (*n* = 2), Staple removal (*n* = 1), Conversion to the total wrist (*n* = 2)
Luegmair and Saffar [25], 2014	10 (7/3)	35 (20–49)	IIIB, IV	Lunarectomy (*n* = 5), preservation (*n* = 5)	8.75 y (1.3–18.6)	Flexion 39 ± 14, Extension 45 ± 15, Radial deviation 16 ± 5, Ulnar deviation 25 ± 6, Grip strength (%) 64 ± 22, VAS 1 ± 0.8	CHR 0.47 ± 0.03, CUDR 0.28 ± 0.03, RSA 52 ± 2	Nonunion (*n* = 1), RS arthritis (*n* = 5) *Reoperations*: PRC (*n* = 1), Radial styloidectomy (*n* = 1), EPL tenolysis (*n* = 1)

N: Number, M-F: Male-Female, y: Year, m: month, ROM: Range of motion, VAS: Visual analogue score, DASH score: Disabilities of Arm, Shoulder, and Hand score, MMW score: Modified Mayo Wrist score, RSA: Radio-scaphoid angle, CHR: Carpal height ratio, LHI: Lunate height index, CUDR: Carpal ulnar distance ratio, NR: Not reported SL: Scapholunate, RS: Radio-scaphoid, IC: Intercarpal, BG: Bone graft, CRPS: Complex regional bone syndrome, EPL: Extensor pollicus longus, (°): Degrees, *: Statistically significant

The debate over lunate management during SCF procedure continues. We suggest that lunarectomy may yield better results by removing necrotic bone tissue with accompanying PCBPs, particularly in the late stages of the disease. This tissue often causes persistent capsular synovitis and pain, which can limit ROM and reduce grip strength. In this regard, ***Charre et al.*** [28] reported no clear advantage of one approach over the other. Conversely, ***Rhee et al.*** [27] and ***Park et al.*** [17] were completely against lunarectomy, suggesting that it increases stress on RSC ligament, potentially leading to carpal-ulnar translation. Due to the nonuniformity of included patients (different KD stages), the limited number of participants, and the short follow-up duration, we cannot conclusively determine whether lunarectomy or lunate preservation is superior. More studies with larger cohorts, comparative analyses, and longer follow-up periods are needed to provide more representative and conclusive findings.

If left untreated, scaphoid rotational malalignment in KD may accelerate pericarpal degenerative changes. ***Minamikawa et al.*** [43] described an ideal RSA in cadaveric study as being between 30° and 57°. Our study found a significant decline in the RSA to 45.88°±7.35° (*P* < 0.00001), which is consistent with a recent meta-analysis [11] with five included seriates [10, 25–28] showing a mean RSA correction to 51.4°. The CHR showed no relevant difference after surgery in our study, with a mean of 0.41 ± 0.08 (*P* = 0.19). This finding was close to those of ***Goyal et al.*** [26], who reported a mean decrease of 0.01 (*P* < 0.05), and ***Luegmair et al.*** [25]. Similarly, ***Charre et al.*** [28] reported a mean decrease in CHR of 0.03 (*P* = 0.05), whereas ***Hegazy et al.*** [24] reported a mean incline in CHR of 0.01 (*P* = 0.71). The mean postoperative Stahl LHI (*n* = 11) was 0.415 ± 0.02, with a non-significant decline of 0.01 among all patients in this study (*P* = 0.33). A stable LHI with a negligible decline of 0.01 can indicate a successful SCF, reducing the risk of further collapse and preserving wrist function. This finding is in line with that reported by ***Hegazy et al.***. (*P* = 0.92) [24]. In contrast, ***Meena et al.*** reported a non-significant increase in LHI by 0.04 ± 0.01 (*P* = 0.48). The mean postoperative modified-CHR in our study was 1.33 ± 0.09, with a significant decrease of 0.09 ± 0.05 (*P* = 0.003). Similar significant decreases in modified-CHR were reported by ***Meena et al.*** (*P* < 0.05) [10] and ***Rhee et al.*** (*P* = 0.01) [27], with mean declines of 0.28 and 0.07, respectively. Conversely, ***Özdemir et al.*** [29] reported nearly identical postoperative ratios (*P* = 1). In this study, we noted a decrease in the average modified-CUDR of 0.8 ± 0.06 (*P* = 0.008). Our findings were consistent with that of ***Rhee et al.****’s* study [27], particularly with lunarectomy (*P* = 0.03). ***Luegmair and Saffar*** [25] also reported a decrease in the mean CUDR from 0.29 ± 0.04 to 0.28 ± 0.03. However, ***Meena et al.*** [10] demonstrated an increase in the mean CUDR from 0.64 ± 0.12 to 0.66 ± 0.14 (*P* < 0.67).

The postoperative ulnar carpal translocation, indicated by a decline in the CUDR, may be attributed to several factors: the inherent progression of Kienböck’s disease as proposed by ***Watson and colleagues*** [44], intraoperative injury to the RSC ligament during procedures like radial styloidectomy or lunarectomy as suggested by ***Nakamura et al.*** [45], and weakened ligamentous integrity due to disease-related synovitis or lunarectomy as described by ***Budoff and colleagues*** [46]. As observed in this study, despite radiographic evidence of ulnar translocation, patients rarely exhibit clinical symptoms. Therefore, we recommend monitoring the modified CUDR or CHR over time rather than relying on a single postoperative assessment, and any changes in these measurements should be correlated with corresponding changes in clinical outcomes.

This study revealed no significant difference in any of the previously mentioned radiographic parameters between stage IIIA and IIIB (*P* > 0.05), postoperatively. Interestingly, there were also no significant differences in these measurements between the lunarectomy and lunate preservation groups (*P* > 0.05), except for the modified-CHR (*P* = 0.002). Similarly, ***Rhee et al.*** [27] demonstrated that the modified-CHR and CUDR significantly declined after lunarectomy, with mean declines of 0.09 ± 0.02 and 0.12 ± 0.08, respectively (*P* = 0.03 and 0.02). However, in the same study, the mean postoperative modified-CHR and CUDR after lunate preservation were 1.40 ± 0.08 and 0.83 ± 0.06, respectively. After lunarectomy, ***Luegmair and Saffar*** [25] reported declines in the means of CHR, CUDR, and RSA by 0.02 ± 0.01, 0.03 ± 0.01, and 18°, respectively. With lunate preservation, the mean decreases were 0.01 ± 0.01, 0.03, and 15°.

This study demonstrated no significant difference in healing time between lunarectomy and lunate preservation (*P* = 0.82), or between stage IIIA and IIIB (*P* = 0.79). Based on these radiological findings, we might suggest that while lunarectomy can offer considerable clinical benefits, it does not alter the radiographic success of the SCF procedure.

Radio-scaphoid arthritis was observed in two patients (8%) in this study, without any clinical complaints. Higher incidences of RS arthritis were reported by ***Park et al.*** [17], ***Charre et al.*** [28], and ***Luegmair et al.*** [25]., with rates of 30.7%, 11.7%, and 50%, respectively. Peri-scaphoid arthritis was also reported by ***Hegazy et al.*** [24] and ***Rhee et al.*** [27]., with incidences of 25% and 40.7%. The lower incidence of RS arthritis in our study may be attributed to the relatively shorter follow-up duration. The reported higher incidence of IC arthritis raises concerns about the longevity of SCF and the potential need for further interventions. Fortunately, the mentioned studies indicated that most patients did not require reoperation, with only 14% of arthritic patients needing revision surgeries. Degenerative changes in the wrist’s radial column following SCF seem inevitable due to the increased joint load and force transmission at the RS and scapho-trapezial-trapezoidal (STT) articulations.

While stage IIIC was not anticipated to directly influence the primary outcomes of our study, its significance within the classification of KD remains noteworthy. Defined by a coronal-oriented lunate fracture and inherent mechanical instability, stage IIIC raises an important clinical consideration whether stage IIIC should be treated similarly to stage IIIB, potentially benefiting from lunarectomy, or preservation.

Our study has several limitations that should be considered when interpreting the findings. First, the retrospective design inherently introduces selection bias. Although we attempted to minimize bias through strict inclusion criteria and appropriate statistical analyses, unmeasured confounders may still have influenced the results. Second, the relatively small sample size reduces the statistical power to detect subtle differences between the study groups. This limitation is particularly relevant when interpreting the non-significant findings. A larger cohort would enhance the reliability and generalizability of our conclusions. Third, the follow-up period was relatively short, which may limit the generalizability of the results and preclude an assessment of long-term outcomes and complications. Moreover, the influence of smoking on healing was not studied due to the lack of available data in the medical records.

A key limitation pertains to the decision-making process for lunate preservation versus lunarectomy, which was made intraoperatively based on the extent of lunate fragmentation, PCBPs, and cartilage degeneration. However, no standardized classification was used to guide this decision objectively, raising the possibility that lunarectomy may have been performed in more severe cases. This lack of a clear classification system introduces a potential selection bias that may have influenced our results. Interestingly, despite this potential bias, the lunarectomy group demonstrated superior pain relief and functional outcomes, suggesting that removing a structurally compromised lunate may provide clinical benefits. Nevertheless, these findings should be interpreted with caution, and future prospective studies with predefined criteria for lunate management are needed to validate our observations.

Furthermore, an important confounding variable in this study is the nonuniformity of the included patients (IIIA vs. IIIB), as disease severity may influence surgical outcomes, pain levels, and functional recovery. Although no significant differences were found, inherent variations in disease progression could still confound the results. Given these limitations, future research with larger cohorts, longer follow-up durations, and objective preoperative criteria for lunate management is necessary to refine treatment strategies for SCF in Kienböck’s disease.

## Conclusion

Our study suggests that SCF is a viable salvage procedure for stage III KD, offering pain relief and functional improvement. While our findings indicate that lunarectomy may provide advantages over lunate preservation, particularly in terms of pain reduction and ROM, these results should be interpreted with caution due to the study’s limitations. The absence of a standardized classification guiding the decision for lunate resection and the potential influence of disease severity on surgical outcomes remain important considerations. Additionally, the non-significant differences in some outcome measures highlight the need for further research. Future prospective studies with larger sample sizes and extended follow-up periods are necessary to better define the role of lunate management in SCF. Understanding how patient-specific factors, such as bone quality and smoking status, affect surgical outcomes will be essential. Ultimately, an individualized approach remains crucial when selecting the optimal surgical strategy for advanced KD, taking into account each patient’s clinical presentation and functional demands.

## Data Availability

No datasets were generated or analysed during the current study.
